# Supplementary material for: Recurrence of Uterine Smooth Muscle Tumor of Uncertain Malignant Potential: A Systematic Review of the Literature
Source: Cancers (Basel). 2022 May 7;14(9):2323. doi: 10.3390/cancers14092323 (PMC9104240; doi:10.3390/cancers14092323)
Supplement: Supplementary file 1 [file cancers-14-02323-s001.zip › cancers-1697951-supplementary.pdf]

Supplementary Table S1. Detailed data synthesis of included studies.

Study	Year	Sample size	Study design	Age at surgery	Parity	Previous CS	Previous pelvic surgery	History of infertility	BMI	Symptoms	Symptoms	Signs	Signs	Ca 125 level	US signs	Largest tumor size (cm)	Myoma localization	Initial surgery	Type pf initial surgery	Morcellation	Mitosis	Atypia	Necrosis	Second surgery	Recurrence	Site of recurrence	Histology of recurrence	Surgical treatment of recurrence	Months of follow up	Pregnancy outcomes	Note
Ning C et al. (27)	2021	16	Case series	34			Unknown	Unknown	Unknown					Unknown				Myomectomy	LPT	No or in a bag	5-9	Moderate	Absent	No	No				78	Yes	
	2021			44			Unknown	Unknown	Unknown					Unknown				Myomectomy	LPS	Unknown	5-9	Mild	Absent	No	No				71	Yes	
	2021			51			Unknown	Unknown	Unknown					Unknown				TAH + BSO	LPT	No or in a bag	5-9	Mild	Absent	No	No				60	No	
	2021			18			Unknown	Unknown	Unknown					Unknown				TAH + BSO	LPT	No or in a bag	5-9	Mild	Absent	No	No				60	No	
	2021			38			Unknown	Unknown	Unknown					Unknown				Isterectomy	LPS	Unknown	5-9	Moderate	Absent	No	No				60	No	
	2021			51			Unknown	Unknown	Unknown					Unknown				Isterectomy	LPT	No or in a bag	5-9	Mild	Absent	No	No				60	No	
	2021			53			Unknown	Unknown	Unknown					Unknown				TAH + BSO	LPS	Unknown	5-9	Moderate	Absent	No	No				60	No	
	2021			40			Unknown	Unknown	Unknown					Unknown				Isterectomy	LPT	No or in a bag	5-9	Moderate	Absent	No	No				60	No	
	2021			46			Unknown	Unknown	Unknown					Unknown				Isterectomy	LPS	Unknown	5-9	Moderate	Absent	No	No				60	No	
	2021			40			Unknown	Unknown	Unknown					Unknown				Myomectomy	LPS	Unknown	5-9	Severe	Absent	No	No				60	Unknown	
	2021			53			Unknown	Unknown	Unknown					Unknown				Myomectomy	LPS	Unknown	5-9	Moderate	Absent	No	No				60	Unknown	
	2021			23			Unknown	Unknown	Unknown					Unknown				Myomectomy	LPS	Unknown	5-9	Mild	Absent	No	No				60	Unknown	
	2021			38			Unknown	Unknown	Unknown					Unknown				TAH + BSO	LPS	Unknown	5-9	Mild	Absent	No	No				60	No	
	2021			52			Unknown	Unknown	Unknown					Unknown				TAH + BSO	LPT	No or in a bag	5-9	Severe	Absent	No	No				60	No	
	2021			38			Unknown	Unknown	Unknown					Unknown				Myomectomy	LPS	Unknown	5-9	Moderate	Present	No	No				60	Unknown	
	2021			56			Myomectomy	Unknown	Unknown					Unknown				Isterectomy	LPS	Unknown	≥10	Moderate	Absent	No	Yes	Pelvis	STUMP	Mass excision	112	No	Free from desease after 112 months
Akad F et al. (28)	2021	1	Case report	50	2	0	Unknown	Unknown	Unknown	Abdominal/pelvic pain	Compression of adjacent organs	Pelvic mass	Anemia	Unknown	a mixt structure consisting of both tissue type and cystic component	19,5		TAH + BSO	LPT	No or in a bag	≥10	Mild	Present	No	No					No	

Yadav G et al. (29)	2021	3	Case series	reproductive age			Unknown	Unknown	Unknown	AUB				Unknown	no suspicion of malignancy	7,0	IM	Myomectomy	LPS	No or in a bag	≥10	Unknown	Unknown	TAH + BSO	No				24	Unknown	
	2021			reproductive age			Unknown	Unknown	Unknown	AUB				Unknown	no suspicion of malignancy	7,0	IM	Myomectomy	LPS	No or in a bag	≥10	Unknown	Unknown	Unknown	No				24	Unknown	
	2021			reproductive age			Unknown	Unknown	Unknown	AUB				Unknown	no suspicion of malignancy	7,0	IM	Myomectomy	LPS	No or in a bag	≥10	Unknown	Unknown	Unknown	No				24	Unknown	
Jang TK et al. (30)	2020	1	Case report	41	1	1	No	No	> 30	Abdominal /pelvic pain	Asthenia	Pelvic mass	Anemia	< 35	30 x 25 cm heterogeneous mass	39,0	IM-SS	Isterectomy	LPT	No or in a bag	≥10	Moderate	Absent	No	No				18	No	
Han AKW et al. (31)	2020	12	Case series	31	0		No	Unknown	Unknown	Abdominal /pelvic pain				Unknown	no suspicion of malignancy	6,5	Unknown	Myomectomy	LPS	Yes	Unknown	Unknown	Unknown	No	No				1	Unknown	
	2020			37	3	0	No	No	Unknown	Dysmenorrhea	Menorrhagia			Unknown	no suspicion of malignancy	2,7	SM	Myomectomy	HSC	No or in a bag	Unknown	Unknown	Unknown	TAH + BSO	Yes	Unknown	STUMP	TAH + BSO	4	Unknown	
	2020			41	0	0	No	Yes	Unknown	Incidentally detected				Unknown	no suspicion of malignancy	3,3	Unknown	Myomectomy	LPS	Yes	Unknown	Unknown	Unknown	No	No				36	Unknown	
	2020			41	0	0	No	Unknown	Unknown	Incidentally detected				Unknown	no suspicion of malignancy	10,0	Unknown	Myomectomy	LPS	Yes	Unknown	Unknown	Unknown	No	No				36	Unknown	
	2020			28	0	0	Myomectomy	No	Unknown			Pelvic mass		Unknown	no suspicion of malignancy	4,7	Unknown	Myomectomy	Unknown	No or in a bag	Unknown	Unknown	Unknown	No	No				40	Unknown	
	2020			44	0	0	No	Unknown	Unknown	Incidentally detected				Unknown	no suspicion of malignancy	4,7	Unknown	Myomectomy	LPS	Yes	Unknown	Unknown	Unknown	No	No				22	Unknown	
	2020			40	0	0	Myomectomy	Unknown	Unknown			Pelvic mass		Unknown	myoma with secondary degeneration	12,6	Unknown	Isterectomy	LPT	No or in a bag	Unknown	Unknown	Unknown	No	No				23	Unknown	
	2020			36	0	0	No	Unknown	Unknown	AUB				Unknown	no suspicion of malignancy	4,8	Unknown	Myomectomy	LPS	No or in a bag	Unknown	Unknown	Unknown	No	No				24	Unknown	
	2020			29	0	0	No	Unknown	Unknown			Pelvic mass		Unknown	no suspicion of malignancy	7,4	Unknown	Myomectomy	LPS	Yes	Unknown	Unknown	Unknown	No	No				24	Yes	
	2020			23	0	0	No	Unknown	Unknown	Dysmenorrhea	Menorrhagia			Unknown	no suspicion of malignancy	6,2	Unknown	Myomectomy	LPS	Yes	Unknown	Unknown	Unknown	No	No				24	No	
	2020			42	0	0	No	Yes	Unknown	Incidentally detected				Unknown	no suspicion of	5,8	Unknown	Myomectomy	LPS	Yes	Unknown	Unknown	Unknown	TAH + BSO	Yes	Unknown	STUMP	TAH + BSO	16	No	

															malignancy																
	2020			41	0	0	No	Yes	Unknown	Incidentally detected				Unknown		4,0	Unknown	Myomectomy	LPS	No or in a bag	Unknown	Unknown	Unknown	No	No				12	Unknown	
Zheng YY et al. (32)	2020	26	Case series	31			Unknown	Unknown	Unknown	Menorrhagia	Abdominal/pelvic pain			Unknown		6,5	IM	Myomectomy	LPT	No or in a bag	5-9	Moderate	Present	No	No				96	Unknown	
	2020			48			Unknown	Unknown	Unknown	Incidentally detected				Unknown		10,0	IM	Myomectomy	LPT	No or in a bag	0-4	Moderate	Present	No	Yes	Uterus	STUMP	Isterectomy	95	Unknown	Free from desease after 95 months
	2020			42			Unknown	Unknown	Unknown	Menorrhagia				Unknown		5,5	IM	Isterectomy	LPT	No or in a bag	0-4	Mild	Present	No	No				91	No	
	2020			42			Unknown	Unknown	Unknown	Incidentally detected				Unknown		3,5	IM	Myomectomy	LPT	No or in a bag	0-4	Mild	Present	No	No				36	Unknown	
	2020			50			Unknown	Unknown	Unknown	Menorrhagia				Unknown		8,0	IM	TAH + BSO	LPT	No or in a bag	0-4	Mild	Present	No	No				76	No	
	2020			44			Unknown	Unknown	Unknown	AUB				Unknown		11,0	IM	Isterectomy	LPT	No or in a bag	0-4	None	Present	No	No				75	No	
	2020			50			Unknown	Unknown	Unknown	AUB				Unknown		11,0	IM	Myomectomy	LPT	No or in a bag	5-9	Severe	Present	No	Yes	Uterus	STUMP	Isterectomy	68	No	Free from desease after 68 months
	2020			34			Unknown	Unknown	Unknown	Compression of adjacent organs				Unknown		7,0	IM	Myomectomy	LPS	Yes	≥10	Mild	Present	No	No				88	Unknown	
	2020			47			Unknown	Unknown	Unknown	Incidentally detected				Unknown		8,0	SS	Isterectomy	LPT	No or in a bag	≥10	None	Present	No	No				85	No	
	2020			39			Unknown	No	Unknown	Abdominal/pelvic pain				Unknown		19,0	SS	Isterectomy	LPT	No or in a bag	5-9	None	Present	No	No				77	No	
	2020			23			Unknown	Unknown	Unknown	Menorrhagia				Unknown		9,0	IM	Myomectomy	LPT	No or in a bag	5-9	Severe	Present	No	Yes	Uterus	STUMP	Isterectomy	74	Unknown	Free from desease after 74 months
	2020			44			Unknown	Unknown	Unknown	Incidentally detected				Unknown		5,3	IM	Isterectomy	LPT	No or in a bag	0-4	Severe	Absent	No	No				73	No	
	2020			38			Unknown	Unknown	Unknown	Menorrhagia				Unknown		8,0	IM	Myomectomy	LPS	Yes	5-9	Mild	Present	No	No				72	Unknown	
	2020			54			Unknown	Unknown	Unknown	Menorrhagia	Dysmenorrhea			Unknown		6,7	SM	TAH + BSO	LPT	No or in a bag	5-9	Mild	Present	No	No				70	No	
	2020			35			Unknown	Unknown	Unknown	Incidentally detected				Unknown		3,7	SS	Myomectomy	LPT	No or in a bag	5-9	Moderate	Present	No	No				69	Unknown	
	2020			48			Unknown	Unknown	Unknown	Incidentally detected				Unknown		2,5	IM	Myomectomy	LPT	No or in a bag	5-9	Severe	Present	No	Yes	Uterus	STUMP	Isterectomy	68	Unknown	Free from desease after 68 months
	2020			43			Unknown	Unknown	Unknown	Menorrhagia				Unknown		10,0	IM	Isterectomy	LPT	No or in a bag	0-4	Severe	Present	No	No				66	No	
	2020			35			Unknown	Unknown	Unknown	Incidentally detected				Unknown		7,5	SM	Myomectomy	LPT	No or in a bag	5-9	Moderate	Present	No	No				67	Unknown	
	2020			45			Unknown	Unknown	Unknown	Menorrhagia				Unknown		6,4	SM	Isterectomy	LPS	Yes	0-4	Severe	Present	No	No				59	No	
	2020			47			Unknown	Unknown	Unknown	Incidentally detected				Unknown		14,0	IM	Myomectomy	LPS	Yes	5-9	None	Present	No	Yes	Uterus	STUMP	Isterectomy	58	Unknown	Free from desease after 58 months
	2020			43			Unknown	Unknown	Unknown	Incidentally detected				Unknown		8,5	SS	TAH + BSO	LPT	No or in a bag	5-9	Moderate	Present	No	No				54	No	

	2020			61			Unknown	Unknown	Unknown	AUB				Unknown		2,5	IM	TAH + BSO	LPT	No or in a bag	0-4	Moderate	Present	No	No				53	No	
	2020			49			Unknown	Unknown	Unknown	Menorrhagia				Unknown		5,3	IM	Isterectomy	LPS	Yes	0-4	Mild	Present	No	No				49	No	
	2020			44			Unknown	Unknown	Unknown	Incidentally detected				Unknown		11,0	IM	Myomectomy	LPS	Yes	0-4	Severe	Present	No	Yes	Uterus	STUMP	Isterectomy	36	Unknown	Free from desease after 36 months
	2020			39			Unknown	Unknown	Unknown	Menorrhagia				Unknown		6,7	IM	Isterectomy	LPS	Yes	0-4	Severe	Absent	No	No				35	No	
	2020			42			Unknown	Unknown	Unknown	Abdominal /pelvic pain				Unknown		15,0	SS	Myomectomy	LPS	Yes	5-9	Severe	Present	No	No				13	Unknown	
Garcia-Sanchez JM et al. (33)	2019	1	Case report	71			No	Unknown	Unknown	Incidentally detected				Unknown		16,0	Unknown	TAH + BSO	LPT	No or in a bag	5-9	Moderate	Present	No	Yes	Other site	LMS	Mass excision		Unknown	Localization of recurrence: right lower limb
	2019	28	Case series	53	6		Unknown	No	> 30			Pelvic mass		< 35		27,0	SS	TAH + BSO	LPT	No or in a bag	0-4	Mild	Present	No	No				44	Unknown	
	2019			58	6		Unknown	No	> 30	AUB				< 35		4,0	IM	TAH + BSO	LPT	No or in a bag	0-4	Moderate	Absent	No	No				6	Yes	
	2019			46	2		Unknown	No	> 30	AUB				< 35		5,5	IM	TAH + BSO	LPS	Unknown	5-9	Moderate	Absent	No	No				8	Unknown	
	2019			75	4		Unknown	No	> 30			Pelvic mass		> 35		12,0	SS	TAH + BSO	LPT	No or in a bag	0-4	Moderate	Absent	No	No				12	Unknown	
	2019			40	1		Unknown	No	< 30			Pelvic mass		< 35		16,0	SM	Isterectomy	LPT	No or in a bag	0-4	Mild	Absent	No	No				12	Unknown	
	2019			35	2		Unknown	No	< 30	AUB				< 35		5,0	IM	Myomectomy	Unknown	Unknown	0-4	Mild	Absent	No	No				14	Unknown	
	2019			43	2		Unknown	No	< 30			Pelvic mass		< 35		11,0	SS	Myomectomy	Unknown	Unknown	0-4	Mild	Present	TAH + BSO	No				17	Unknown	
Karatasli V et al. (34)	2019			50	3		Unknown	No	< 30	AUB				< 35		8,0	SS	TAH + BSO	LPT	No or in a bag	0-4	Mild	Absent	No	No				18	Unknown	
	2019			40	0		Unknown	Unknown	< 30			Pelvic mass		> 35		12,0	IM	Isterectomy	LPT	No or in a bag	5-9	Moderate	Absent	No	No				48	Yes	
	2019			52	2		Unknown	No	< 30			Pelvic mass		< 35		9,0	IM	TAH + BSO	LPS	Unknown	0-4	Mild	Absent	No	No				61	Unknown	
	2019			42	2		Unknown	No	> 30	AUB				< 35		8,5	IM	Myomectomy	Unknown	Unknown	0-4	Mild	Absent	TAH + BSO	No				47	Unknown	
	2019			43	2		Unknown	No	< 30	AUB				< 35		7,0	IM	Isterectomy	LPT	No or in a bag	5-9	Mild	Absent	No	No				24	Unknown	
	2019			54	3		Unknown	No	> 30	AUB				< 35		8,0	IM	TAH + BSO	LPT	No or in a bag	0-4	Moderate	Present	No	Yes	Pelvis	LMS		62	Unknown	Died of desease (lyposarcoma) after 62 months
	2019			29	1		Unknown	No	< 30	Incidentally detected				< 35		10,0	IM	Myomectomy	Unknown	Unknown	0-4	Mild	Present	No	No				39	Unknown	
	2019			48	2		Unknown	No	< 30			Pelvic mass		< 35		6,0	IM	TAH + BSO	LPT	No or in a bag	0-4	Moderate	Absent	No	No				6	Unknown	

	2019			41	2		Unknown	No	> 30	AUB				< 35		2,0	IM	Isterectomy	LPS	Unknown	5-9	Mild	Absent	No	No				48	Unknown	
	2019			39	2		Unknown	No	< 30	AUB				< 35		6,0	IM	Myomectomy	Unknown	Unknown	0-4	Moderate	Absent	Isterectomy	No				52	Unknown	
	2019			42	2		Unknown	No	< 30	AUB				< 35		7,5	IM	Isterectomy	LPT	No or in a bag	5-9	Moderate	Absent	No	No				47	Unknown	
	2019			39	2		Unknown	No	< 30	AUB				< 35		22,0	IM	Isterectomy	LPT	No or in a bag	5-9	Moderate	Absent	No	No				56	Unknown	
	2019			45	3		Unknown	No	> 30	AUB				< 35		5,0	IM	Isterectomy	LPS	No or in a bag	0-4	Moderate	Absent	No	No				23	Unknown	
	2019			46	3		Unknown	No	> 30	AUB				< 35		5,0	IM	TAH + BSO	LPT	No or in a bag	0-4	Severe	Absent	No	No				31	Unknown	
	2019			34	1		Unknown	No	< 30	AUB				< 35		5,0	IM	Myomectomy	Unknown	Unknown	0-4	Severe	Absent	No	No				44	Yes	
	2019			49	1		Unknown	No	> 30	AUB				< 35		4,0	IM	TAH + BSO	LPS	Unknown	5-9	Severe	Absent	No	No				5	Unknown	
	2019			48	2		Unknown	No	> 30	AUB				< 35		5,0	IM	TAH + BSO	LPT	No or in a bag	5-9	Mild	Absent	No	No				15	Unknown	
	2019			43	3		Unknown	No	< 30			Pelvic mass		< 35		6,0	IM	Isterectomy	LPT	No or in a bag	5-9	None	Absent	No	No				163	Unknown	
	2019			43	2		Unknown	No	> 30	AUB				< 35		6,5	IM	Isterectomy	LPT	No or in a bag	0-4	Moderate	Absent	No	No				57	Unknown	
	2019			31	2		Unknown	No	< 30			Pelvic mass		< 35		4,5	IM	TAH + BSO	LPT	No or in a bag	0-4	Moderate	Absent	No	No				180	Unknown	
	2019			38	2		Unknown	No	< 30	AUB				< 35		3,0	SS	Myomectomy	Unknown	Unknown	0-4	Moderate	Absent	TAH + BSO	No				131	Unknown	
Ha HI et al. (35)	2018	19	Case series	41	3		Unknown	No	Unknown	Incidentally detected				Unknown		7,0	Unknown	Isterectomy	LPT	No or in a bag	0-4	Moderate	Absent	No	No					Unknown	
	2018			30	0		Unknown	No	Unknown	Incidentally detected				Unknown		8,0	Unknown	Myomectomy	Unknown	Unknown	0-4	Mild	Absent	No	No					Unknown	
	2018			47	2		Unknown	No	Unknown	Incidentally detected				Unknown		9,0	Unknown	TAH + BSO	LPT	No or in a bag	0-4	Moderate	Absent	No	No					Unknown	
	2018			48	3		Unknown	Unknown	Unknown	Incidentally detected				Unknown		12,0	Unknown	Isterectomy	LPT	No or in a bag	5-9	Mild	Absent	No	No					Unknown	
	2018			40	2		Unknown	Unknown	Unknown	Incidentally detected				Unknown		12,0	Unknown	Isterectomy	LPT	No or in a bag	≥10	Mild	Absent	No	No					Unknown	
	2018			49	2		Unknown	Unknown	Unknown	Incidentally detected				Unknown		4,0	Unknown	Isterectomy	Unknown	Unknown	5-9	Mild	Absent	No	No					Unknown	
	2018			48	3		Unknown	Unknown	Unknown	Incidentally detected				Unknown		9,5	Unknown	Myomectomy	Unknown	Unknown	0-4	Mild	Absent	No	No					Unknown	
	2018			49	2		Unknown	Unknown	Unknown	Incidentally detected				Unknown		12,0	Unknown	Myomectomy	Unknown	Unknown	5-9	Mild	Present	TAH + BSO	No					Unknown	
	2018			30	0		Unknown	Unknown	Unknown	Incidentally detected				Unknown		8,0	Unknown	Myomectomy	Unknown	Unknown	5-9	Moderate	Absent	No	No					Unknown	
	2018			46	1		Unknown	Unknown	Unknown	Incidentally detected				Unknown		10,0	Unknown	Isterectomy	LPT	No or in a bag	≥10	Mild	Absent	No	No					Unknown	
	2018			39	1		Unknown	Unknown	Unknown	Incidentally detected				Unknown		10,0	Unknown	Myomectomy	Unknown	Unknown	0-4	Moderate	Present	No	Yes	Pelvis	STUMP	Mass excision		Unknown	
	2018			49	1		Unknown	Unknown	Unknown	Incidentally detected				Unknown		7,0	Unknown	Isterectomy	LPT	No or in a bag	0-4	Moderate	Absent	No	No					Unknown	
	2018			40	2		Unknown	Unknown	Unknown	Incidentally detected				Unknown		13,0	Unknown	Myomectomy	Unknown	Unknown	≥10	Mild	Absent	Isterectomy	No					Unknown	
	2018			32	1		Unknown	Unknown	Unknown	Incidentally detected				Unknown		6,0	Unknown	Myomectomy	Unknown	Unknown	5-9	Moderate	Absent	No	No					Unknown	

	2018			33	0		Unknown	Unknown	Unknown	Incidentally detected				Unknown		6,0	Unknown	Myomectomy	Unknown	Unknown	5-9	Moderate	Absent	No	No					Yes	
	2018			45	1		Unknown	Unknown	Unknown	Incidentally detected				Unknown		10,0	Unknown	Isterectomy	LPT	No or in a bag	5-9	Moderate	Absent	No	No					Unknown	
	2018			49	2		Unknown	Unknown	Unknown	Incidentally detected				Unknown		6,0	Unknown	Isterectomy	LPS	Unknown	0-4	Mild	Absent	No	Yes	Pelvis	LMS	Mass excision	24	Unknown	
	2018			35	0		Myomectomy	Unknown	Unknown	Incidentally detected				Unknown		7,0	Unknown	Myomectomy	Unknown	Unknown	0-4	Mild	Absent	No	No					Unknown	
	2018			28	0		Unknown	Unknown	Unknown	Incidentally detected				Unknown		26,0	Unknown	Myomectomy	Unknown	Unknown	5-9	Mild	Absent	No	No					Unknown	
Huges L et al. (36)	2018	1	Case report	20	0		No	No	Unknown	Menorrhagia	Abdominal /pelvic pain	Pelvic mass	Anemia	Unknown	Subserosal fibroids at hte fundus of the uterus measuring 20 x 18 cm	20,0	SS	Myomectomy	LPT	No or in a bag	5-9	Moderate	Absent	No	No					Unknown	
Oda H et al. (37)	2018	1	Case report	40			Unknown	Unknown	Unknown	Abdominal /pelvic pain		Anemia		> 35	Peritoneal bleeding	22,0	Unknown	Myomectomy	LPT	No or in a bag	5-9	Mild	Absent	No	Yes	Pelvis	LMS	Debulking + CTx	40	Unknown	Died of desease 40 months after initial surgery
Basaran D et al. (38)	2018	21	Case series	28			Unknown	Unknown	Unknown	Incidentally detected				Unknown		15,0	Unknown	Myomectomy	LPT	No or in a bag	5-9	Moderate	Absent	No	No				33	Unknown	
	2018			63			Unknown	Unknown	Unknown	Incidentally detected				Unknown		2,5	Unknown	TAH + BSO	LPT	No or in a bag	0-4	Severe	Absent	No	No				78	Unknown	
	2018			42			Unknown	Unknown	Unknown	Incidentally detected				Unknown		5,5	Unknown	TAH + BSO	LPT	No or in a bag	0-4	Mild	Present	No	No				8	Unknown	
	2018			64			Unknown	Unknown	Unknown	Incidentally detected				Unknown		18,0	Unknown	TAH + BSO	LPT	No or in a bag	0-4	None	Present	No	No				22	Unknown	Non desease-related death
	2018			48			Unknown	Unknown	Unknown	Incidentally detected				Unknown		8,0	Unknown	TAH + BSO	LPT	No or in a bag	0-4	Mild	Present	No	No				56	Unknown	
	2018			52			Unknown	Unknown	Unknown	Incidentally detected				Unknown		8,0	Unknown	TAH + BSO	LPT	No or in a bag	5-9	Mild	Present	No	No				99	Unknown	
	2018			47			Unknown	Unknown	Unknown	Incidentally detected				Unknown		4,0	Unknown	TAH + BSO	LPT	No or in a bag	0-4	None	Present	No	No				132	Unknown	
	2018			37			Unknown	Unknown	Unknown	Incidentally detected				Unknown		1,8	Unknown	Isterectomy	LPT	No or in a bag	0-4	Mild	Present	No	No				39	Unknown	
	2018			40			Unknown	Unknown	Unknown	Incidentally detected				Unknown		4,0	Unknown	Isterectomy	LPT	No or in a bag	0-4	Moderate	Present	No	No				68	Unknown	
	2018			39			Unknown	Unknown	Unknown	Incidentally detected				Unknown		5,5	Unknown	Myomectomy	LPT	No or in a bag	0-4	None	Present	No	No				10	Unknown	
	2018			37			Unknown	Unknown	Unknown	Incidentally detected				Unknown		11,0	Unknown	Myomectomy	LPT	No or in a bag	0-4	Moderate	Present	No	No				12	Unknown	
	2018			20			Unknown	Unknown	Unknown	Incidentally detected				Unknown		15,0	Unknown	Myomectomy	LPT	No or in a bag	0-4	None	Present	No	No				52	Unknown	
	2018			28			Unknown	Unknown	Unknown	Incidentally detected				Unknown		9,0	Unknown	Myomectomy	LPT	No or in a bag	0-4	None	Present	No	No				88	Unknown	
	2018			51			Unknown	Unknown	Unknown	Incidentally detected				Unknown		20,0	Unknown	Isterectomy	LPT	No or in a bag	0-4	None	Present	No	No				18	Unknown	
	2018			44			Unknown	Unknown	Unknown	Incidentally detected				Unknown		9,0	Unknown	Isterectomy	LPT	No or in a bag	0-4	None	Present	No	No				54	Unknown	
	2018			43			Unknown	Unknown	Unknown	Incidentally detected				Unknown		6,5	Unknown	Isterectomy	LPT	No or in a bag	0-4	None	Present	No	No				56	Unknown	

	2018			51			Unknown	Unknown	Unknown	Incidentally detected				Unknown		7,0	Unknown	TAH + BSO	LPT	No or in a bag	0-4	Moderate	Absent	No	No				74	Unknown	
	2018			52			Unknown	Unknown	Unknown	Incidentally detected				Unknown			Unknown	TAH + BSO	LPT	No or in a bag	0-4	Mild	Present	No	Yes	Pelvis	LMS	Mass excision	95	Unknown	Died of desease after 95 months
	2018			51			Unknown	Unknown	Unknown	Incidentally detected				Unknown		9,0	Unknown	TAH + BSO	LPT	No or in a bag	0-4	Mild	Present	No	Yes	Pelvis	LMS	Mass excision	69	Unknown	Free from desease after 154 months
	2018			28			Unknown	Unknown	Unknown	Incidentally detected				Unknown		9,0	Unknown	Myomectomy	LPT	No or in a bag	0-4	None	Present	No	Yes	Uterus	STUMP	Myomectomy	154	Unknown	Free from desease after 45 months
	2018			38			Unknown	Unknown	Unknown	Incidentally detected				Unknown			Unknown	Myomectomy	LPT	No or in a bag	0-4	None	Present	No	Yes	Uterus	LMS	TAH + BSO	45	Unknown	Free from desease after 69 months
Bacanakgli BH et al. (39)	2017	6	Case series	48	5		Unknown	No	Unknown	Menorrhagia		Pelvic mass		Unknown	Well-defined heterogeneous mass	3,5	IM	TAH + BSO	LPT	No or in a bag	0-4	Mild	Present	No	No				120	Unknown	
	2017			44	6		Unknown	No	Unknown	Menorrhagia				Unknown	Well-defined heterogeneous mass	8,0	SM	TAH + BSO	LPT	No or in a bag	≥10	Moderate	Present	No	No				119	Unknown	
	2017			40	2		Unknown	No	Unknown	Menorrhagia				Unknown	Well-defined hyperechoic mass	7,0	IM	Myomectomy	Unknown	Unknown	5-9	Moderate	Absent	No	No					Unknown	
	2017			43	4		Unknown	No	Unknown	Menorrhagia				Unknown	Well-defined hyperechoic mass	20,0	IM	TAH + BSO	LPT	No or in a bag	5-9	Moderate	Absent	No	No				38	Unknown	
	2017			52	4		Unknown	No	Unknown	Menorrhagia				Unknown	Well-defined hyperechoic mass	10,0	IM	TAH + BSO	LPT	No or in a bag	5-9	Mild	Absent	No	No				29	Unknown	
	2017			24	0		Unknown	Unknown	Unknown	Menorrhagia				Unknown	Well-defined hyperechoic mass	8,0	IM	Myomectomy	Unknown	Unknown	5-9	Moderate	Absent	No	Yes	Unknown	STUMP	Myomectomy	11	Unknown	
Bogani G et al. (40)	2016	1	Case report	37	2		Unknown	No	Unknown	Menorrhagia				Unknown		13,0	SS	Myomectomy	LPS	Yes	Unknown	Unknown	Unknown	TAH + BSO	Yes	Abdomen	STUMP	Mass excision		Unknown	
Sakuragy Y et al. (41)	2016	1	Case report	68			Unknown	Unknown	Unknown	Incidentally detected				Unknown			Unknown	Isterectomy	Unknown	Unknown	5-9	Unknown	Unknown	No	Yes	Lung	STUMP	Mass excision	2	Unknown	recurrence sites: lung and scalp skin
Kalogiannidis I et al. (42)	2016	5	Case series	46			Unknown	Unknown	Unknown	Menorrhagia				Unknown		6,0	Unknown	TAH + BSO	LPT	No or in a bag	0-4	Severe	Absent	No	No				132	Unknown	
	2016			43			Unknown	Unknown	Unknown	Menorrhagia				Unknown		5,0	Unknown	Isterectomy	LPT	No or in a bag	0-4	Severe	Absent	No	No				108	Unknown	
	2016			42			Unknown	Unknown	Unknown	Menorrhagia				Unknown		4,0	Unknown	Isterectomy	LPT	No or in a bag	0-4	Severe	Absent	No	No				72	Unknown	
	2016			43			Unknown	Unknown	Unknown	Incidentally detected				Unknown		4,0	Unknown	Myomectomy	LPS	Unknown	5-9	Severe	Absent	No	No				28	Unknown	
	2016			26			Unknown	Unknown	Unknown	Incidentally detected				Unknown		6,5	Unknown	Myomectomy	LPT	No or in a bag	0-4	Severe	Absent	No	No				15	Unknown	

Campbell JE et al. (43)	2015	1	Case report	41	4	1	Unknown	No	Unknown	Menorrhagia	Abdominal/pelvic pain			Unknown		10,0	IM	Myomectomy	LPT	No or in a bag	5-9	Mild	Present	Isterectomy	No				48	Yes	
Mowers EL et al. (44)	2015	5	Case series	38			Unknown	Unknown	Unknown	Incidentally detected				Unknown			Unknown	TAH + BSO	LPS	No or in a bag	Unknown	Unknown	Unknown	No	Yes	Pelvis	STUMP	Mass excision	3	Unknown	
	2015			40			Unknown	Unknown	Unknown	Incidentally detected				Unknown			Unknown	Isterectomy	LPS	Yes	Unknown	Unknown	Unknown	No	Yes	Abdomen	STUMP	Mass excision	24	Unknown	
	2015			50			Unknown	Unknown	Unknown	Incidentally detected				Unknown			Unknown	TAH + BSO	LPS	Yes	Unknown	Unknown	Unknown	No	Yes	Abdomen	STUMP	Mass excision	35	Unknown	
	2015			47			Unknown	Unknown	Unknown	Incidentally detected				Unknown			Unknown	Isterectomy	LPS	Yes	Unknown	Unknown	Unknown	No	Yes	Pelvis	STUMP	Mass excision	28	Unknown	
	2015			50			Unknown	Unknown	Unknown	Incidentally detected				Unknown			Unknown	Myomectomy	LPT	Yes	Unknown	Unknown	Unknown	No	Yes	Pelvis	STUMP	TAH + BSO	32	Unknown	
Zhang R et al. (45)	2015	1	Case report	42			Unknown	Unknown	Unknown	Incidentally detected				Unknown			Unknown	TAH + BSO	LPT	No or in a bag	0-4	Severe	Absent	Unknown	No					Unknown	sign: fever
Dall'Asta A et al. (46)	2014	5	Case series	44	0		Unknown	Unknown	Unknown	AUB	Abdominal/pelvic pain	Anemia		Unknown		12,0	Unknown	Isterectomy	LPT	No or in a bag	Unknown	Severe	Unknown	No	No				81	Unknown	
	2014			51	2	2	Unknown	No	Unknown			Pelvic mass		< 35		12,0	Unknown	TAH + BSO	LPT	No or in a bag	Unknown	Severe	Present	No	No				78	Unknown	
	2014			49	1		No	No	Unknown	AUB				Unknown		2,5	Unknown	TAH + BSO	LPT	No or in a bag	Unknown	Severe	Absent	No	No				33	Unknown	
	2014			45	0		Unknown	Unknown	Unknown	Incidentally detected				Unknown		14,0	Unknown	Myomectomy	LPT	No or in a bag	0-4	Severe	Absent	No	No				19	Unknown	
	2014			48	2		No	No	Unknown	AUB				Unknown			IM	Myomectomy	LPS	Unknown	0-4	Mild	Absent	TAH + BSO	No				3	Unknown	
Kotsopoulos IC et al. (47)	2012	1	Case report	51	0		D&C	Unknown	Unknown	Menorrhagia				Unknown		5,0	IM	Isterectomy	LPT	No or in a bag	0-4	Moderate	Absent	No	Yes	Lung	STUMP		11	Unknown	Died of disease
Seidman MA et al. (7)	2012	3	Case series	24			Unknown	Unknown	Unknown			Pelvic mass		Unknown		6,6	Unknown	Myomectomy	LPS	Yes	Unknown	Unknown	Unknown	No	Yes	Unknown	STUMP		12	Unknown	
	2012			41			Unknown	Unknown	Unknown			Pelvic mass		Unknown		10,0	Unknown	Myomectomy	LPS	Yes	Unknown	Unknown	Unknown	No	Yes	Unknown	STUMP		13	Unknown	
	2012			45			Unknown	Unknown	Unknown			Pelvic mass		Unknown		8,7	Unknown	Isterectomy	LPS	Yes	Unknown	Unknown	Unknown	No	Yes	Unknown	STUMP		28	No	
Hsieh J et al. (48)	2012	1	Case report	37	0	0	No	No	Unknown	Compression of adjacent organs		Pelvic mass		Unknown	MR: characterized a 4.6×4.2×4.0 cm mass located within the anterior vaginal wall with mild homogeneous enhancement	4,6	Unknown	Myomectomy	LPT	No or in a bag	0-4	Moderate	Absent	Unknown	No					Unknown	
Vilos AG et al. (49)	2012	2	Case	35	1	0	No	No	< 30	AUB		Pelvic		Unknown	Multiple myomas, with	9,8	IM	Myomectomy	LPT	No or in a bag	5-9	Severe	Absent	Isterectomy	No				60	Unknown	

			series									mass			the largest located posteriorly and measuring 9.7x9.8x8.8 cm, and another that memeasuring 3.6x2.3x2.3 cm																
	2012			39	0	0	No	Yes	< 30	Abdominal /pelvic pain		Pelvic mass		Unknown	Multiple uterine myomas, with the 2 largest measuring 8.6x7.4x7.2 cm and 6.0x6.0x5.3 cm		IM-SS	Myomectomy	LPT	No or in a bag	0-4	Mild	Present	Isterectomy	No				40	Unknown	
Canciani GN et al. (50)	2012	1	Case report	48			No	Unknown	Unknown	Compression of adjacent organs		Pelvic mass		Unknown			Unknown	Isterectomy	LPT	No or in a bag	0-4	Mild	Absent	No	Yes	Pelvis	STUMP	Mass excision	288	No	
Hong IH et al. (51)	2011	1	Case report	49			Unknown	Unknown	Unknown	Abdominal /pelvic pain		Pelvic mass		Unknown	CT showed a 10x17x19 cm mass at the posterior side of the uterus	17,0	SS	TAH + BSO	LPT	No or in a bag	5-9	Moderate	Absent	Unknown	No					No	
Müller Vranjes A et al. (52)	2011	1	Case report	37			Unknown	Unknown	Unknown	Abdominal /pelvic pain		Pelvic mass		Unknown		20,0	Unknown	Myomectomy	LPT	No or in a bag	Unknown	Unknown	Unknown	Isterectomy	No					Unknown	
Gezginc K et al. (53)	2011	1	Case report	42	0		No	No	Unknown	Menorrhagia	Dysmenorrhea	Pelvic mass		< 35	9x7 cm heterogeneous mass in the uterus cavity	9,0	SM	TAH + BSO	LPT	No or in a bag	0-4	Mild	Present	No	No				24	No	
Yoon BS et al. (54)	2011	1	Case report	28	0	0	No	No	Unknown	Menorrhagia		Pelvic mass		Unknown	Multiple intramural leiomyomata, including 3 measuring 8,0x7.5 cm, 6.8x5.1 cm, and 6.0x5.8 cm.	8,0	IM	Myomectomy	LPT	No or in a bag	5-9	Severe	Absent	No	Yes	Uterus	LMS	Isterectomy	36	No	Alive after 36 months of follow up
IP PP et al. (55)	2009	16	Case series	50			Unknown	Unknown	Unknown	AUB				Unknown		4,0	IM	TAH + BSO	Unknown	Unknown	0-4	Moderate	Present	No	Yes	Pelvis	LMS	Mass excision	51	No	Alive 74 months afet mass excision

	2009			52			Unknown	Unknown	Unknown	AUB		Anemia		Unknown		7,5	IM	TAH + BSO	Unknown	Unknown	0-4	Moderate	Absent	No	No					No	
	2009			39			Unknown	Unknown	Unknown	AUB		Pelvic mass		Unknown		5,8	IM	Isterectomy	Unknown	Unknown	5-9	Moderate	Present	No	Yes	Pelvis	LMS	Debulking + CTx	15	No	Alive 40 months after Debulking
	2009			39			Unknown	Unknown	Unknown	AUB				Unknown		5,0	SM	TAH + BSO	Unknown	Unknown	5-9	Mild	Present	No	No					No	
	2009			56			Unknown	Unknown	Unknown			Pelvic mass		Unknown		10,0	IM	TAH + BSO	Unknown	Unknown	0-4	Moderate	Present	No	No					No	
	2009			64			Unknown	Unknown	Unknown			Pelvic mass		Unknown		6,0	IM	TAH + BSO	Unknown	Unknown	0-4	Moderate	Present	No	No					No	
	2009			46			Unknown	Unknown	Unknown	AUB				Unknown		12,0	SM	TAH + BSO	Unknown	Unknown	0-4	Severe	Present	No	No					No	
	2009			50			Unknown	Unknown	Unknown	AUB		Anemia		Unknown		0,7	IM	Myomectomy	Unknown	Unknown	0-4	Severe	Present	Isterectomy	No					No	
	2009			48			Unknown	Unknown	Unknown			Pelvic mass		Unknown		17,0	IM	TAH + BSO	Unknown	Unknown	5-9	Severe	Present	No	No					No	
	2009			40			Unknown	Unknown	Unknown			Pelvic mass		Unknown		8,5	IM	TAH + BSO	Unknown	Unknown	5-9	Severe	Present	No	No					No	
	2009			59			Unknown	Unknown	Unknown	AUB				Unknown		18,0	IM	TAH + BSO	Unknown	Unknown	0-4	Severe	Present	No	No					No	
	2009			25			Unknown	Unknown	Unknown			Pelvic mass		Unknown		16,0	IM	TAH + BSO	Unknown	Unknown	5-9	None	Present	No	No					No	
	2009			49			Unknown	Unknown	Unknown			Pelvic mass		Unknown		11,0	IM	TAH + BSO	Unknown	Unknown	5-9	None	Present	No	No					No	
	2009			46			Unknown	Unknown	Unknown	AUB				Unknown		5,0	SS	Myomectomy	Unknown	Unknown	5-9	Severe	Present	No	No					Unknown	
	2009			50			Unknown	Unknown	Unknown	AUB				Unknown			SM	TAH + BSO	Unknown	Unknown	0-4	Severe	Absent	No	No					No	
	2009			44			Unknown	Unknown	Unknown	AUB		Anemia		Unknown		5,0	SM	Myomectomy	Unknown	Unknown	≥10	Severe	Present	Isterectomy	No					No	
Berretta R et al. (56)	2008	3	Case series	44	2	0	No	No	Unknown	Compression of adjacent organs		Pelvic mass		Unknown	Multiple intramural leiomyomas and one 8x3x8 cm subserous anterior myoma.	8,0	SS	Isterectomy	LPT	No or in a bag	0-4	Mild	Present	No	No				60	No	
	2008			35	0	1	No	No	Unknown	Compression of adjacent organs		Pelvic mass		Unknown	Subserosal, pedunculated myoma	16,0	SS	Myomectomy	LPT	No or in a bag	≥10	Mild	Absent	No	No				12	No	
	2008			37	2	0	No	No	Unknown	AUB				Unknown	Leiomyoma measuring 8x3x7 cm	10,0	IM	Isterectomy	LPT	No or in a bag	5-9	Severe	Absent	No	Yes	Lung	STUMP	Mass excision	60	No	
Huang SE et al. (57)	2008	1	Case	47	2	0	No	No	Unknown	Compression of		Pelvic		> 35		20,0	SS	TAH + BSO	LPT	No or in a bag	5-9	Severe	Absent	No	No				24	No	

			report							adjacent organs		mass																			
Amant F et al. (58)	2005	1	Case report	48	2	0	No	No	Unknown	AUB				Unknown		7,0	SS	TAH + BSO	LPT	No or in a bag	0-4	None	Present	No	Yes	Pelvis	LMS	Mass excision	48	No	
Shapiro A et al. (59)	2004	1	Case report	46	8	0	No	No	Unknown	Menorrhagia		Anemia		Unknown	Single, submucous leiomyoma measuring 3,5x4x4,5 cm	4,0	SM	TAH + BSO	Unknown	Unknown	≥10	Severe	Absent	No	Yes	Other site	LMS	Mass excision	51	No	51 months after isterectomy: fracture of the right humerus (LMS) --> Mass excision. 12 months later: lung nodules (metastasis from LMS)

Abbreviations: CS: cesarean section; BMI: body mass index (kg/m^2); AUB: abnormal uterine bleeding; US: ultrasounds; MRI: magnetic resonance; CT: computered tomography; IM: intramural; SS: subserosal; SM: submucosal; TAH: total abdominal hysterectomy; BSO: bilateral salpingo-oophorectomy; D&C: dilation&curettage; LPS: laparoscopy; LPT: laparotomy; HSC: hysteroscopy; LMS: leiomyosarcoma; LM: leyomioma; CTx: chemotherapy.
